# Abdominal Bulging Due to Abdominal Muscle Palsy Secondary to Herpes Zoster: A Report of a Rare Case

**DOI:** 10.7759/cureus.76440

**Published:** 2024-12-26

**Authors:** Atsushi Hiramoto, Ryo Nagase, Yosuke Nakata, Yuki Arai, Takashi Sato

**Affiliations:** 1 Department of Gastroenterology, Nasu Red Cross Hospital, Otawara, JPN

**Keywords:** abdominal bulging, abdominal muscle palsy, abdominal pseudohernia, herpes zoster, varicella zoster virus

## Abstract

A 59-year-old man visited our hospital for examination of left-sided abdominal bulging. About a week earlier, he had developed an abdominal skin rash and was diagnosed with herpes zoster. Computed tomography excluded intra-abdominal organic disease and true hernia. We diagnosed the patient with abdominal muscle palsy, i.e., abdominal pseudohernia, secondary to varicella zoster reactivation. Abdominal pseudohernia is a rare complication of herpes zoster but is not life-threatening. Nevertheless, physicians should keep this clinical entity in mind when examining a patient presenting with abdominal wall bulging.

## Introduction

Gastroenterologists, gastroenterological surgeons, and general practitioners often examine patients who present with bulging in the abdominal wall, with or without abdominal pain. Emergent etiologies, such as intra-abdominal bleeding or gastrointestinal perforation, typically cause global abdominal bulging [1]. The majority of localized abdominal bulging is caused by hernias, including inguinal, umbilical, and incisional hernias [2], but it may rarely be caused by an undescended testicle, a harmless hematoma, or a lipoma. In even rarer circumstances, it may be an intra-abdominal mass such as a cancerous tumor [3]. Therefore, keeping a broad differential diagnosis is essential. Imaging modalities, such as X-ray, ultrasonography, CT, and MRI, are used in addition to physical examination for assessment [3]. Herein, we describe our experience with a patient who developed abdominal bulging caused by abdominal muscle palsy, i.e., abdominal pseudohernia [4], secondary to varicella zoster reactivation. Although this is an extremely rare presentation, zoster should be kept in mind as part of the differential diagnosis when encountering a patient with abdominal wall bulging.

## Case presentation

**Figure 1:**
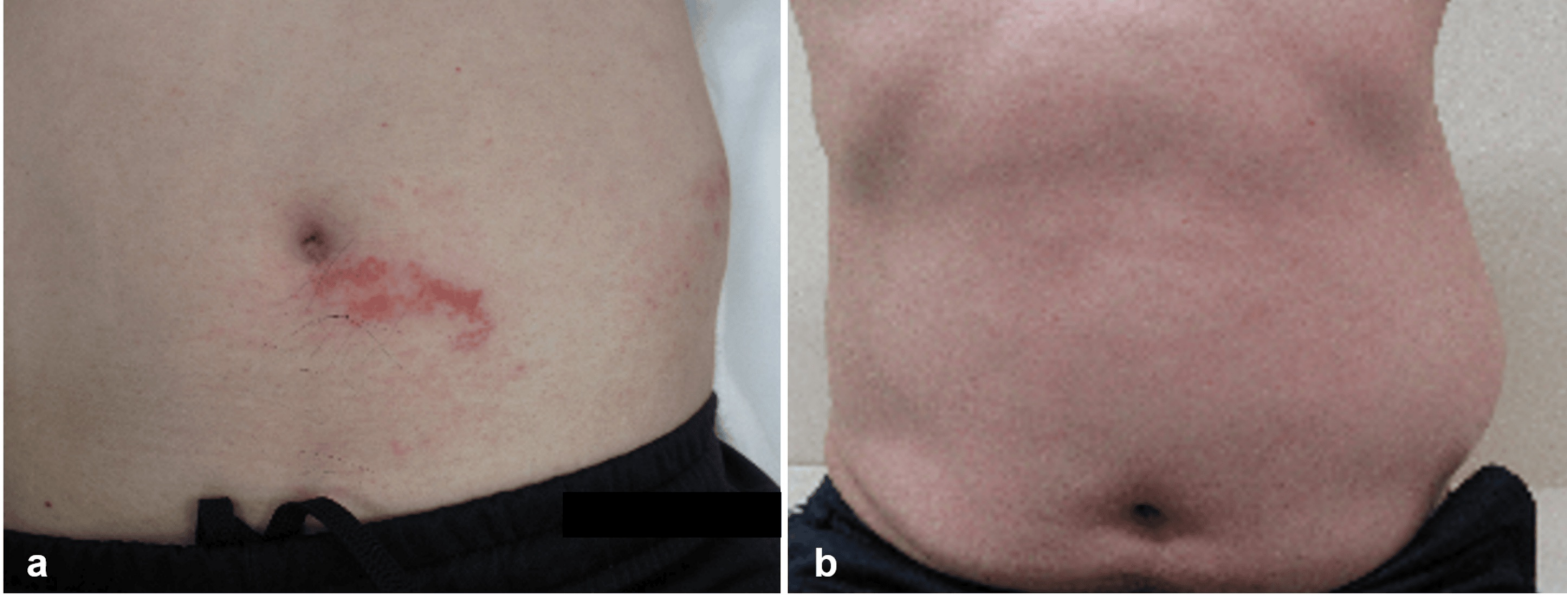
Photographs. (a) At the first hospital visit, a red rash is observed at the lower left side of the navel, near the abdominal bulge. (b) Three months later, the abdominal bulging persists.

**Figure 2:**
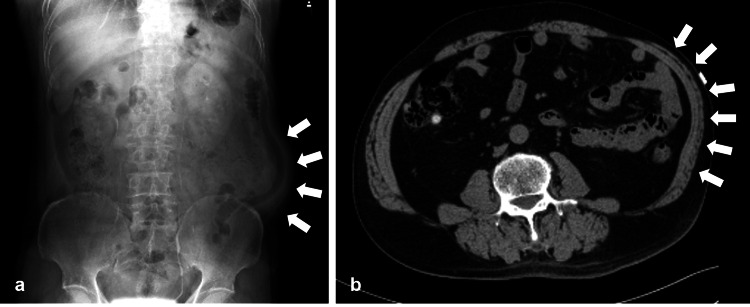
(a) Plain abdominal X-ray: The profile of the left-sided abdominal wall bulging is evident (arrows), but no detailed structure is visible. (b) Abdominal computed tomography without contrast (horizontal section): Thinning of the left-sided abdominal muscles is seen (arrows), but there is no lesion in the intra-abdominal space.

A 59-year-old man was referred to Nasu Red Cross Hospital by a local practitioner for examination of left-sided abdominal bulging. About a week earlier, he had developed an abdominal skin rash with blisters, which had been diagnosed as herpes zoster in the T-10 dermatome of the left lower abdomen and had been treated with the helicase-primase inhibitor amenamevir (400 mg/day for 7 days) and methylcobalamin (1500 µg/day). A few days prior to presentation, a soft bulging appeared in his left lower abdomen. On his first hospital visit, this bulging was painless and nontender to palpation, although the patient had dermatalgia caused by herpes zoster. The bulging expanded with the Valsalva maneuver. In addition, a red rash was observed at the lower left side of the navel, near the abdominal bulge (Figure 1a). Laboratory blood analysis was within normal limits. A plain abdominal X-ray showed the profile of the left-sided abdominal bulging, but no detailed structure was confirmed (Figure 2a). On the horizontal section imaging of an abdominal CT scan, there was thinning of the left abdominal muscles but no organic lesion (e.g., mass) in the intra-abdominal space, and a true hernia was also ruled out (Figure 2b). Using this information, we diagnosed the cause of the abdominal bulging as abdominal muscle palsy, i.e., abdominal pseudohernia, secondary to herpes zoster. Since the bulging was asymptomatic and did not interfere with the patient’s daily life, we decided to observe and to treat only for postherpetic neuralgia (methylcobalamin, 1500 µg/day). The abdominal bulging persisted for more than 3 months, but there was no sign of enlargement, so the patient continued under observation (Figure 1b)

## Discussion

We describe herein our experience with a patient who had abdominal bulging caused by abdominal muscle palsy secondary to herpes zoster. Our initial differential diagnosis included intra-abdominal organic disease and hernia. We made the final diagnosis using CT scan imaging and the patient's recent history of herpes zoster.

Herpes zoster is a clinical syndrome caused by the reactivation of latent varicella zoster virus in the ganglia of the cranial nerves or dorsal root ganglia. It is characterized by a skin rash that spreads along the dermatomes. The virus affects sensory nerves, from the ganglia to the corresponding skin dermatome, resulting in dermatalgia. Herpes zoster may also involve the adjacent motor nerve branches, causing facial nerve palsy known as Ramsay Hunt syndrome [5]. A review article in 1972 reported that 5.0% of herpes zoster patients experienced motor paralysis. Ramsay Hunt syndrome was seen in 1.7% of all patients with herpes zoster and 34.4% of patients with motor paresis. Abdominal muscle palsy was evident in only 0.17% of all patients with herpes zoster and 3.3% of patients with motor paresis [6].

Abdominal muscle palsy secondary to herpes zoster causes a type of abdominal bulging called abdominal pseudohernia [3,7-9]. The pseudohernia differs from a true hernia in that there is no actual muscular disruption: all muscular and fascial layers remain intact [10]. In addition to herpes zoster motor paralysis, abdominal pseudohernia has been reported in association with a variety of syndromes involving neuropathy or denervation, including diabetes mellitus [11], disc herniation [12,13], rib fractures [10], or surgical injury [14].

Very recently, Chiew YR and Pawa C [15] conducted a literature search for abdominal pseudohernia due to herpes zoster using PubMed over a 20-year period, from 2001 to 2021. In their report, a total of 34 individuals, including their own patient, were reviewed. The median patient age at presentation was 71.5 years (range, 43-82 years). Twenty-seven patients (79.4%) were male. The median interval from the appearance of the skin rash to the onset of abdominal bulging was 14 days (range, 1-60 days). Interestingly, there were 4 patients (12.5%) in whom abdominal bulging occurred prior to the onset of the herpes zoster rash. The thoracic dermatome most commonly affected was T-11 (n = 20; 66.7%), while T-10 was involved in our patient. During the median follow-up period of 3 months, the majority of patients (n = 21; 70%) recovered completely, and 5 patients (16.7%) showed significant improvement. Regarding the prognosis, Chernev I and Dado D [7] reported that 79.3% of patients eventually recovered and the mean recovery period is 4.9 months. Therefore, our patient continued under observation, although he was still affected three months later.

Because of the self-limited nature and good prognosis of abdominal pseudohernia due to herpes zoster, physicians should avoid unnecessary diagnostic studies and procedures. Although imaging modalities, including ultrasonography, CT scan, and MRI, are useful to exclude intra-abdominal disease and true hernia, the diagnosis of pseudohernia is relatively easy based on the unique characteristics of the abdominal bulging, the relevant medical history, and the presence of a rash. Thinning of the abdominal muscles on CT, as seen in our patient, is a typical finding of this condition; this thinning may also be seen on MRI [16]. The use of MRI may also be indicated to check for herpes zoster-induced radiculoneuritis of the posterior nerve root and the spinal nerve [16]. In addition, electrodiagnostic examination, such as intercostal nerve conduction study or electromyography of the abdominal wall muscles, could be helpful for diagnosing radiculoneuropathy [7], although these procedures are not essential to diagnose abdominal pseudohernia due to herpes zoster. However, one should not exclude pseudohernia simply because there is no rash, since the bulging precedes the herpetic rash in over 10% of patients [15]. It is important for all physicians to keep this clinical entity in mind when examining a patient who presents with abdominal wall bulging.

## Conclusions

Abdominal pseudohernia is a rare complication of herpes zoster. It can be diagnosed based on a history of herpes zoster and imaging modalities such as CT or MRI. However, in rare cases, abdominal bulging may precede the skin rash. Although it is not a life-threatening complication, physicians should keep this clinical entity in mind when examining a patient presenting with abdominal wall bulging.
